# Messenger-based assessment of empathic accuracy in couples’ smartphone communication

**DOI:** 10.1186/s40359-025-02483-9

**Published:** 2025-02-21

**Authors:** Philipp Steinebach, Miriam Stein, Knut Schnell

**Affiliations:** 1 Department of Psychiatry and Psychotherapy, University Medical Centre, Georg-August Universität Göttingen, Göttingen, Germany; 2 Institute of Psychology, Universität Heidelberg, Heidelberg, Germany; 3 Department of Psychiatry and Psychotherapy, University Medical Centregeorg-August Universität Göttingenand Psychiatry Clinic Asklepios, Göttingen, Germany

**Keywords:** Empathic accuracy, Affect, Messenger, Couple relationships

## Abstract

**Background:**

How accurate are empathic judgments of couples in smartphone messenger communication? Are judgments influenced by the level of experience with messengers and communication frequency?.

**Objectives:**

The current preregistered study introduced a novel ecological assessment method and a privacy by design approach to study couples’ empathic accuracy in a messenger context.

**Methods:**

Data from *N* = 102 participants (51 couples) was used to investigate how accurate judgments of partners’ affect map their partners’ actual affect.

**Results:**

Our results demonstrate tracking accuracy and assumed similarity bias in reciprocal empathic judgments of affective valence and arousal during social messenger texting. A small moderation effect for experience with text messengers was found, indicating that partners with higher experience have a higher bias of assumed similarity when rating their partner’s valence. A small moderation effect for communication frequency confirms that higher messenger communication frequency is associated with more accurate judgments of arousal.

**Conclusion:**

These results point to the reciprocal action of accuracy and bias in couples’ messenger communication and the distinct influences of experience and usage. The feasibility and further application of the ecological messenger-based assessment of couples’ empathic accuracy in interpersonal research are discussed.

**Supplementary Information:**

The online version contains supplementary material available at 10.1186/s40359-025-02483-9.

## Background

Changes in communication patterns of couples due to increasing online and messenger-based communication may alter reciprocal understanding [1]. Within the dynamics of couple relationships, reciprocal understanding or empathic accuracy is of key interest in both research and interventions or counseling for couples [2]. Having the ability to grasp mental states and personal traits in your partner appears to be beneficial for relationships [3]. Thus, investigating messenger-based understanding in couples is of importance to understand the complex dynamics of couples’ wellbeing and adapt interventions for couples [4].

However, the assessment of the complex dynamic of understanding and misunderstandings in couples has proven to be difficult. Laboratory-based assessment (e.g. [5]) can be invasive and the delay between relevant situations and ratings can lead to biases. Experience sampling methods (ESM; [6, 7]) have the drawback of predefined assessment times, often resulting in different assessment contexts (e.g. partner present vs. not present). Consequently, the present research adds to the heterogeneity of assessment approaches to study empathic accuracy in couples by using a novel messenger-based assessment to examine two main research questions. First, we try to replicate findings of tracking accuracy and assumed similarity bias in the context of couples’ messenger communication. Second, we investigate whether experience with messengers and communication frequency act as moderators of empathic accuracy and bias.

### Messenger communication in couples

Given the growing importance of online communication and instant messaging, research has addressed patterns and issues in couples’ web-based interaction and identified positive (e.g. [8]) as well as negative (e.g. [9]) outcomes. Differential effects for computer-mediated communication modes (e.g. text messages, email, phone call, or video call) can be assumed [10]. Experimental studies find evidence for less beneficial effects of text-message compared with face-to-face support provision [8] and reduced positive interpersonal behaviors in text messaging in comparison to face-to-face conflict interactions [9]. Openness in text messaging has even been linked to lower relationship satisfaction and discussing the state of relationships via text messages is associated with higher face-to-face conflict [11, 12]. Young couples tend to discuss potentially relationship threatening topics via messenger [13], which can lead to a higher risk of conflict. However, in an experimental study, no differences between text messaging and face-to-face interactions for conflict resolution outcomes were found [14]. Given the heterogeneity of findings, underlying processes should be addressed. Avoidance of miscommunication or the ability to accurately judge one another, termed empathic accuracy [15, 16], could partially explain issues due to text messaging in couples’ everyday life. Generally, a higher risk of miscommunication is present in online communication [17], which can be partially attributed to neutrality and negativity biases that lead to distorted evaluations of online messages [18]. Miscommunication in couples can also be increased in the case of messaging during other activities, the unavailability of nonverbal information, the use of acronyms and punctuation as well as technical issues [19]. Surprisingly, a series of experimental studies on the effects of the modality of communication (voice-only vs. videoconference) in strangers found a higher accuracy in the judgment of a dialog partner’s affect in voice-only communication. The author explained this effect by the low levels of intimacy in participants [20]. So far, only one study has examined empathic accuracy and bias in couples’ phone communication. In an event-contingent recording study, accuracy of affiliation ratings of self and partner were higher in face-to-face communication compared with unspecified phone-mediated interactions, including texting or voice calls [1]. Our study aims to extend these findings and investigate accuracy and bias in couples’ text messages in the context of a novel messenger app implemented as an assessment tool.

### Experience with messengers

Different stages of a relationship are associated with differences in couples’ texting behavior [12]. Partners tend to develop similarities in their communication style throughout initial stages of their relationship [21]. However, research on relationship length as a moderator of accuracy and bias for various constructs (e.g. personality traits, attitudes, behavior), has produced mixed results [22, 23]. Empathic accuracy does not clearly improve over the progress of a relationship [24]. Regarding partners’ age, no clear association can be found with increases in empathic accuracy, with some studies arguing that a cognitive decline but higher knowledge and motivation in empathic accuracy is present in older age (see [25] for a review). A study on the effect of presence of a partner on empathic accuracy found higher empathic accuracy in younger couples when partner was present but no differences between older and younger couples when the target partner was absent [26]. In turn, the usage of smartphone messengers and experience in smartphone mediated communication are negatively correlated with age [27, 28]. In a recent study on the smartphone use in the German general population, different types of smartphone users are identified regarding their frequency, skills, and activities. These user types were related to age and educational attainment but existed throughout all age groups [29]. Experience in text messaging will likely impact skills, such as emoji usage in text messages, that can potentially facilitate understanding [30]. Though correlated with age and relationship length, experience with messengers is hypothesized to be independently related to accuracy and bias in couples’ empathic judgments of each other.

### Accuracy and bias in couples

Empathic accuracy, or the accurate representation of momentary affective states of the partner is seen as important interpersonal ability in relationships [31]. Meta-analytic results point to a weak but robust connection between empathic accuracy and relationship satisfaction [3]. However, recent studies point towards a complex relation and emphasize the importance of differential analysis of valence of partner affect (positive vs. negative), differences in actor and partner effects, gender effects, as well as differences between direct outcomes and outcomes over a period [3, 32]. Furthermore, empathic accuracy should be differentiated from the feeling of being understood, which shows heterogeneous associations with actual accuracy [33]. Motivated inaccuracy, a theoretical conceptualization of the dynamics of empathic accuracy, postulates that accuracy can be reduced in the presence of potentially relationship threatening topics or information [34]. This theory could not be fully confirmed in recent studies [35, 36]. However, research in groups with high psychological symptoms did show the postulated dynamic [37, 38]. Overall, it is to assume that accuracy of affect relates to more positive than negative effects for the process of reciprocal understanding in couples’ [39] and miscommunication might lead to lower relationship satisfaction [17].

To disentangle accuracy and bias in couples, the truth and bias model differentiates between mean-level bias, tracking accuracy, and bias of assumed similarity [40]. Tracking accuracy or correlational accuracy refers to the congruence of partners’ rating of mental states over fluctuations and represents the truth force in the judgment [40], which is a robust effect in couples [41]. In turn, a mean-level bias, or a general tendency towards a more negative or positive perception of the partner is frequent in various relationship stages [42] and a bias towards a more positive evaluation shows associations with relationship stability [23, 43]. Bias of assumed similarity or the tendency to infer a partner’s mental state based on perceiver’s own mental states, is another robust effect in couples’ perception [40, 44, 45]. It is in line with findings on the neurobiological foundations of cognitive empathy, which suggest that empathic judgements are based on self-referential simulations of others’ affective states [46]. In general, accuracy and bias play an important part in couples’ relationship processes and have the function to simultaneously manage motives to understand one’s partner, see the partner as someone positive and feel similar to a partner [23, 41, 42]. Thus, it is necessary to address aspects of accuracy and bias in research on couples’ affective judgments.

### Assessment of accuracy and bias

The most frequently used assessment methods of empathic accuracy in the past decades, such as the dyadic interaction paradigm [5], are laboratory based [3]. Laboratory based methods have the drawbacks of (a) motivated accuracy by direct instructions to be as accurate as possible and the subsequent checking of accuracy, which can reduce ecological validity of assessments [47]; (b) delayed ratings of affect after a target situation ended, could lead to substantial memory bias and can be biased by the general capacity to infer affective or mental states in one’s partner [36, 48, 49]; and (c) restricted possibilities to examine different affective or motivational states, since concepts have to be present in the targets using experimental situations, such as the induction of couple conflicts via relationship threatening topics (for an overview of the validity of laboratory and daily life methods, see [50] and [49]). To date, only few studies on empathic accuracy in couples were conducted using daily life measures of empathic accuracy, which in contrast to laboratory-based assessments, can assess natural interactions using frequent ratings over the course of multiple days via mobile devices or short reports [32, 36, 51]. Daily life measures, also called experience sampling or intensive repeated measures in naturalistic settings, themselves differ in the method applied to prompt ratings of experiences. Interval-contingent and signal-contingent prompting refer to a time interval (e.g. day) or signal (e.g. several prompts randomly throughout a day), which initiates ratings, whereas event-contingent recordings use the occurrence of predefined events (e.g. couple conflict) to initiate ratings, which are related to these events (e.g. partner’s feelings) [52]. A benefit of event-contingent methods is the higher immediacy of ratings, which reduces cognitive biases. Studies have found substantial correlations between real-time measures and ratings given shortly after target situations [49]. In the context of couple communication, it is however likely that different contexts between couple interaction and assessment lead to biased judgments of partner affect and mental states. Partners can differ greatly in their affective states during and shortly after conflict [53]. Assessments after couple interactions likely lead to a change in perspective, an effect which is frequently used in couple interventions to resolve fights through the facilitation of less emotionally charged interpretations, which help to understand the partner’s viewpoint [54]. It can also be regarded as a case of the mental or actual location updating effect [55], leading to altered memories after a change of the context. Furthermore, as postulated in the Event Horizon Model [56], differences in the perception of event boundaries, that are essential in event-contingent ratings, where participants have to decide for themselves when an event (e.g. communication sequence) ended, can lead to memory biases [57]. One study compared effects of intuitive processes and systematic thoughts on empathic accuracy in strangers and found that systematic thought shows a robust connection with higher levels of empathic accuracy [58]. A recent study extends on these findings by proposing fast and slow empathic accuracy processes, which account for different levels of accuracy and bias in couples, depending on the response time of ratings [59]. Also, motivational processes can influence empathic accuracy [60, 61] and deliberate or systematic empathic accuracy processes might be used only when a motivation to do so is present [62]. In sum, the assessment of empathic accuracy can be biased when the assessment context allows for extensive systematic thinking. However, couple interactions that are relevant for dissatisfaction are often based on fast intuitive processes and patterns of recurring dynamics which are driven by high levels of emotional arousal, such as the demand-withdraw interaction pattern [63–65]. Overall, real-time assessment should therefore take place in the same context as the interaction and be timed during or as early as possible after an interaction. Consequently, we aim at the assessment of fast and intuitive rather than slow and systematic processes of empathic accuracy.

### The present study

The present study proposes a novel assessment method using a smartphone messenger application. Research questions were focused on the accuracy of participants’ judgments of their partner’s affect and on the bias by the perceivers’ current own affect within an interaction. We also examined experience with smartphone messengers and communication frequency as moderators of accuracy and bias. The present study extends the existing research on couples’ empathic accuracy and text messaging behavior in various ways. We applied a smartphone messenger-based assessment system, which allows the time efficient rating of own and partner’s valence and arousal of affect during text messaging interactions within the same application. This methodological procedure broadens the range of methods, used to date in studies on couples’ accuracy and bias [52]. Also, to our knowledge, no study has yet addressed experience with messengers or communication frequency as moderators in couples’ accuracy and bias in text messages.

All hypotheses, procedures and analysis were preregistered [66] and all deviations from this preregistration were highlighted.^1^

In line with previous research, we hypothesized that partners would show accuracy in their judgment of partners’ affect [23]. In detail, we propose that individuals’ judgments of their partners’ valence and arousal of affect are positively connected to their partners’ self-referential ratings of their own valence and arousal of affect, while controlling for assumed similarity or bias of partner’s rating by perceiver’s own affect. In addition, we expected that experience with messengers as well as communication frequency of text messages act as moderators of empathic accuracy: we expected a higher experience with messengers to be correlated with a higher empathic accuracy, indicated by a higher tracking accuracy. Since we expected higher levels of confidence in the rating of text message interactions in participants with high levels of experience with messengers, a lower reliance on own affect is expected. The same moderation effect is expected for communication frequency of chat messages. Due to the novelty of the assessment method, we had no hypotheses about the magnitude of the expected effects.

## Method

Measures and analysis scripts are available at [68]. Additional information on the rating interface and the messenger-based assessment matrix of affect can be found in the additional file 1.

### Participants

Due to the novelty of our assessment method, no a priori information about effect sizes existed and power estimations were based on general considerations of dyadic data analysis and correlations[69, 70],^2^ resulting in a planned sample size of 200 couples. These considerations are in line with recommended sample sizes of 175 to detect average sized effects in psychological research on the couple level with 80% power (*r* = 0.21; [72]).

Participants were recruited from the community via online advertisements and classified ads. The initial incentives for participation were a personalized relationship feedback, based on their app use, and the free use of the app during the study. Participants were advised that their personal relationship feedback would be more meaningful if they rated their partners frequently. After four months of the study phase, an initially not preregistered financial incentive was included to improve recruitment. Participants were offered to receive a €20 voucher after completion of the study. The present research is based on a sample of 51 couples (102 individuals). Although we did not meet our desired sample size, the present research falls in line with the general recommendation of 50 level two units (e.g. couples) for multilevel research [73].

To be eligible for participation, couples had to be in a committed relationship for at least 6 months, both partners had to be at least 18 years of age and speak German fluently. Inclusion criteria were not preregistered. 121 couples completed the initial online questionnaire and downloaded the app. We lowered our preregistered inclusion criteria to 5 complete messenger-based ratings per couple to increase sample size, resulting in a total of 51 couples and a dropout of 57%. Wilcoxon tests showed that completion of the messenger-based assessment study protocol was associated with lower age (*p* < 0.001, effect size *r* = 0.17), higher experience with messengers (*p* < 0.01, effect size *r* = 0.13), but not associated with relationship length (*p* = 0.124, effect size *r* = 0.08). Most participants were between 26 and 30 years old (*IQR* = 1, range: 18–25 to 61–65 years), in a relationship with their partner for 4–10 years (*IQR* = 1, range: less than one year to more than 10 years), and 57 percent of participants reported living with their partner. Male gender was reported by 47% of the participants (45% female, 5% diverse and 3% did not report their gender) and 88% reported a mixed-sex relationships (8% same-sex, 2% diverse, 2% did not report their gender).

### Procedure

Potential participants were guided to a website, which informed about the study. After giving online-consent, participants could download the app and pair with their partner using a personalized study code. Instructions were given to participants in a step-by-step online and in-app introduction to using the app. These instructions included the rating systems, the use of the app, the purpose of the messenger-based assessment, and privacy as well as security information. We made sure that participants fully understood the affective rating system and the used valence arousal matrix by including test questions at the end of the introduction. Participants completed online surveys before and after the messenger-based assessment phase. The completion of the initial survey automatically unlocked the chat and rating functions of the app. All participants used their own Android or iOS smartphone. The software used is ‘DIADIC’ and was developed by equalia (www.equalia.de) as a tool for communication training and research in couples. The app offers chat communication for couples via text-messages and specific symbols for affect and interpersonal motives. During the first 10-day interval, ratings for partners were hidden between partners. Furthermore, the communication of affect- and motive-symbols was disabled in the chat to avoid sharing affect and motives before the ratings. In the directly following 10-day interval, ratings were disclosed and the communication of affect via symbols was enabled. All analysis were based on the data of the first 10-day interval. We used an event-contingent sampling scheme, and the ratings could be completed self-initiated. To start the rating procedure, one partner clicks on a button inside the chat and completes all ratings in an in-app rating interface. We used single-item ratings of self and other to reduce workload and distraction. The other partner is notified that a rating procedure has started and is advised to complete the corresponding ratings via notifications in the chat. Automatic prompts were sent to the chat when couples exchanged at least two messages per partner in five minutes. Prompts advised couples to initiate the real-time rating procedure. We set a limit of five daily prompts and intervals between prompts of at least one hour. Participants were advised to rate their own and their partner’s affect and interpersonal motives in the present moment. In the original version of the procedure, both partners are presented with a result score indicating the correspondence of their judgements. To avoid training effects, the scores were hidden in the assessment phase of the study. Participants were also instructed that affect items refer to their momentary experience, but that their item responses should be independent from the actual assessment itself. This means that answers should be based on the feeling in that moment, during the chat and not feelings that arise due to rating. Participants were advised to rate at least five times per day. At the end of the messenger-based assessment, participants were guided to the final online survey, debriefed via server-generated messages in their app, and received their automatically generated couple report as well as their financial compensation, if applicable.

**Fig. 1:**
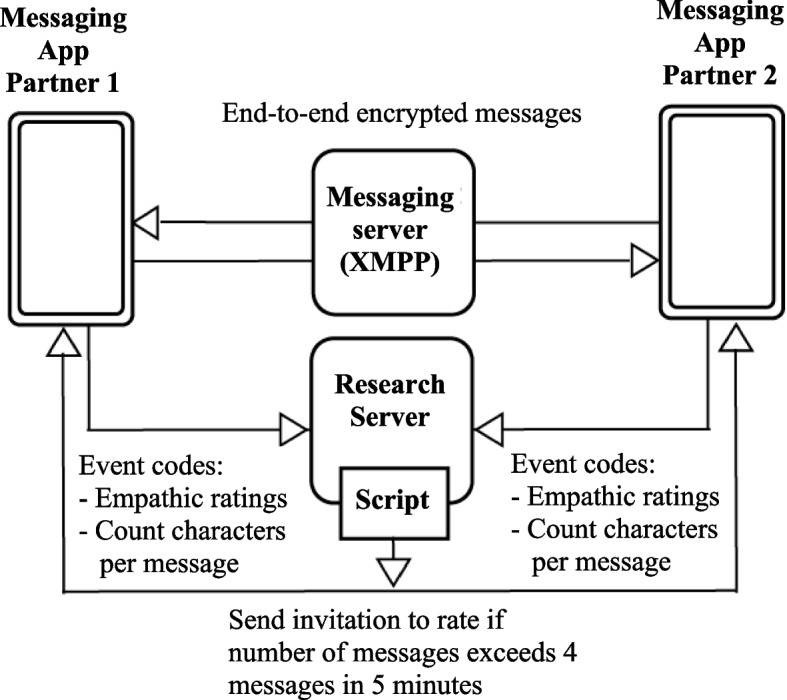
Technical assessment process and privacy by design approach of the assessment app. Note. Event codes of both partners enter the research server (center). The XMPP server (top) contains the end-to-end encrypted messages, which are separated from the research server. Research server scripts (bottom) guide participants through the assessment procedure

To ensure privacy of participants, a privacy by design approach was implemented [74]. No personally identifiable information was collected, and communication was solely based on personalized study codes and online or in-app instructions. The messenger app was based on the Extensible Messaging and Presence Protocol (XMPP) and used a dedicated XMPP server. Chat content was generally end-to-end encrypted. Prompts to start real-time assessments were automatically triggered by a Python [75] study script executed on a backend study server system, which was separated from the XMPP server. Only the affective ratings and the number of characters in text messages as well as the time of the event and the participants study ID were sent from the messenger app to the study server system. The study server consisted of a combination of server applications namely ngnix, rabbitmq, django as well as a library of Python scripts implementing the study system. All servers were installed in separate docker containers on a virtual machine (vm-ware) based at the IT center of the University of Goettingen. Figure 1 gives an overview of the technical assessment process and the privacy by design approach of the assessment app.

### Measures

#### Messenger-based assessment of affect

The messenger-based assessment questions consisted of two ratings of affect (one rating for self, one rating for partner).^3^ We used a single-item instrument based on the valence-arousal model of affect (following Posner, Russell, & Peterson [76]). Each value is represented using specific symbols inside a 7 × 4 matrix. Based on one rating in the matrix, two scores, one for valence and one for arousal were extracted. The assessment of self and partner is estimated to take two minutes in total to be completed. The 51 couples reported in total 960 jointly reported messenger-based interactions (Mean per couple = 18,82, *SD* = 12,85, range = 5–61).

#### Experience with messengers

Experience with messengers was assessed by asking participants to indicate their individual experience with messenger-apps (How experienced are you personally with the usage of messenger apps?) on a 5-point (1–5; *very little – very much*) scale (*M* = 4.30, *SD* = 0.90, range = 1–5). Partners differed in their individual experience with messengers (*r* = 0.16), so that individual scores were used in the subsequent analysis.

#### Communication frequency of text messages

The total number of sent text messages in the app per person (*M* = 129.76, *SD* = 179.84, *range* = 0–888) and the mean text volume or character length per sent message in the app per person (*M* = 35.58, *SD* = 25.90, range = 0–181) were used to evaluate communication frequency of text messages. Since we had no differential hypotheses for number of messages and text volume, a composite measure for communication frequency was constructed by averaging the z-standardized scores of the number of messages and message volume (*M* = 0, *SD* = 0.72, range = −1.31–3.02). Partners differed in their individual communication frequency (*r* = 0.63), so that individual scores were used in the subsequent analysis.

## Results

### Analytic strategy

To analyze mean-level bias, tracking accuracy, and bias of assumed similarity, we used the dyadic multilevel Truth and Bias Model of judgment (T&B model [40]) and conducted separate models for valence and arousal of affect. The T&B model allows for the concurrent analysis of mean-level bias and tracking accuracy, while controlling for one another [77]. The applied model accounts for nonindependence in dyadic data, using a two-level model. The first level refers to within-person variation and the second level refers to between-person variation or across couples. Following Stern & West [77], perceiver’s judgment of the partner’s affect (e.g. valence and arousal) on each rating is explained by an intercept, the partner’s actual reported affect, the perceiver’s own self-reported affect, and random error. We were interested in general levels of mean-level bias, so we centered the outcome and predictors on the grand-mean (mean across ratings and across partners) of the partner’s actual affect. Since predictors and outcome are assessed on the same scale, the intercept represents the mean-level bias, with a significant negative intercept indicating underestimation of partner’s general affect and a significant positive intercept indicating overestimation of partner’s general affect. Due to the centering of partner’s actual reported momentary affect on its grand-mean, it represents the truth force or tracking accuracy, since it indicates how strongly perceivers can track fluctuations in partners’ affect. Finally, centering of perceiver’s own affect on the grand-mean of partner’s actual self-referential momentary affect leads to the interpretability of bias of assumed similarity[40, 77].^4^ Partner’s reported affect and perceiver’s own affect were entered as fixed effects. Since we included non-heterosexual couples, diverse gender participants and participants, who did not indicate their gender, dyads are seen as indistinguishable. A compound symmetry variance–covariance matrix was specified and random effects were estimated for each dyad. Therefore, dummy variables were included for the two arbitrary partners and constraints were forced on the variance–covariance matrix at the level of the random effects, to make model parameters equal for both partners [77]. The analysis of indistinguishable dyads, though sparsely applied in couple research (for an example using structural equation modeling see [44]), has the benefit of higher power because information is pooled across partners [79]. Contrary to our preregistration, we did not explore indirect accuracy due to convergence issues. A test of our preregistered inclusion criteria of a maximum duration of 15 min between partners’ ratings revealed that 235 rating procedures exceeded this criterium. In 40 cases the evaluation of rating times was not possible due to technical issues. To avoid a further drop in power, all ratings remained in the analysis.

For the moderation analysis, experience with messengers and communication frequency were entered as moderators in two separate analysis and grand-mean centered on their own mean. Interaction terms between the moderator and partner’s actual momentary affect as well as an interaction between the moderator and perceiver’s own affect are incorporated in the model. Interaction terms indicate the change in tracking accuracy and assumed similarity bias due to a one-unit change in the moderator variable [40, 77]. Additionally, we calculated simple slopes for the significant moderation effects. Therefore, we recentered moderators, resulting in two variables with moderator levels at one SD above and below zero. We then calculated interaction effects between moderator variables at different levels and TAB variables.

Due to prior reports of an impact of relationship length on accuracy and bias [24], we included relationship length and age as covariates. We also controlled for understanding of symbols in the app, to ensure that effects were not due to misunderstanding of the novel procedures. Gender was not included as a covariate, since we included a diverse category and allowed for participants to not indicate their gender, which made the gender variable not meaningful. Also, our low sample did not allow for group comparisons between gender and group interaction subgroups. Contrary to our preregistration, we made several changes to the included covariates. First, we did not control for day of assessment to reduce model complexity. Second, since we found significantly lower experience with messengers in dropouts, we additionally included the number of ratings provided as a covariate to avoid bias by participation frequency. Finally, in the moderation analysis of communication frequency, we included an additional covariate to control for the use of other text messenger apps. Participants indicated after the study to what extent they used additional messenger apps to communicate with their partner during the study.

All T&B analysis were conducted using SAS/STAT version 15.2 [80] and were based on the PROC MIXED syntax provided by Stern & West [77]. Data handling and descriptive statistics were conducted in R version 4.1.3 [81].

### Descriptive statistics

Initially we examined the distribution of predictors and outcome variables using histograms. Means, medians, standard deviations, and correlations of messenger-based assessment variables are presented in Table 1. Pairwise correlations for indistinguishable dyads are computed following the double-entry method [78, 82]. The correlation coefficient of valence and prediction of valence was significantly different from the correlation coefficient of arousal and prediction of arousal (*Z* = −7.08, *p* < 0.001), indicating a difference between valence and arousal judgment accuracies. Within-couple correlations of all but arousal judgments were significant, indicating the non-independence of the dyadic data.

**Table 1:** Means, standard deviations, and pairwise correlations with confidence intervals

Variable	*M*	*Mdn*	*SD*	*Range*	1	2	3	4
1. Arousal judgment	1.66	2	0.82	0 to 3	-.00			
					[-.07, .06]			
2. Actual arousal of partner	1.70	2	0.84	0 to 3	.07*	.07*		
					[.01, .13]	[.01, .13]		
3. Valence judgment	0.89	1	1.77	−3 to 3	-.05	-.10**	.41***	
					[-.01., .11]	[.04, .16]	[.36, .46]	
4. Actual valence of partner	1.13	2	1.69	−3 to 3	-.04	-.12***	.37***	.28***
					[-.02., .10]	[.06., .18]	[.09, .64]	[.31, .43]

*N* = 51 couples, 102 participants, 960 jointly reported ratings. *M*, Mdn, and *SD* are used to represent mean, median, and standard deviation, respectively. Correlations below the diagonal represent pairwise correlations for indistinguishable dyads [78, 82]. Correlations on the diagonal refer to within-couple correlations. Values in square brackets indicate the 95% confidence interval for each correlation. * indicates *p* < .05. ** indicates *p* < .01. *** indicates *p* < .001

### Accuracy and bias of affect

The first T&B model with the variance–covariance matrix proposed by Stern & West [77] failed to converge and constraints on the variance–covariance matrix were iteratively reduced for all models to simplify the covariance structure until convergence criteria were met following recommendation by Kiernan, Tao, & Gibbs [83]. Of the initial twelve constraints on the variance–covariance matrix, eight parameters for the valence model and four parameters for the arousal model remained in the analysis.^5^

Consistent with our hypothesis, we found significant levels of tracking accuracy for valence and arousal indicating that couples were accurate in their judgment of their partner’s affect. We also found significant levels of assumed similarity bias of own affect on partner’s valence or arousal. Partners relied on their own affect in the judgment of their partner’s affect. In both models, the influence of tracking accuracy was substantially lower than the influence of assumed similarity bias. On average, participants neither over- nor underestimated their partner’s valence and arousal, represented by a non-significant negative mean-level bias. In the arousal model, none of the covariates had a significant effect and in the valence model, only age was positively related to judgment of partner’s affect. All covariates remained in the model.^6^ Results of the valence and arousal models are presented in Tables 2 and 3.

**Table 2:** Mean-level bias, tracking accuracy, and projection bias in the judgment of partner’s valence

Effects	Estimate	*SE*	*t*	*p*	*CI*
Mean-level bias	−0.232	0.117	−1.98	0.055	[−0.469; 0.005]
Tracking accuracy	0.260	0.028	9.40	< .0001	[0.205; 0.316]
Projection bias	0.421	0.026	15.98	< .0001	[0.368; 0.475]
Age	−0.283	0.058	−4.88	< .0001	[−0.399; −0.168]

*N* = 51 couples, 102 participants, 960 jointly reported ratings. Solution for fixed effects of multilevel truth and bias analysis. Estimates refer to unstandardized effects. Not displayed are non-significant covariates (relationship length, number of ratings and understanding of symbols in the app). CI = 95%

**Table 3:** Mean-level bias, tracking accuracy, and projection bias in the judgment of partner’s arousal

Effects	Estimate	*SE*	*t*	*p*	*CI*
Mean-level bias	−0.021	0.027	−0.77	0.450	[−0.078; 0.036]
Tracking accuracy	0.088	0.026	3.39	0.001	[0.037; 0.140]
Projection bias	0.290	0.032	8.97	< .0001	[0.226; 0.354]

*N* = 51 couples, 102 participants, 960 jointly reported ratings. Solution for fixed effects of multilevel truth and bias analysis. Estimates refer to unstandardized effects. Not displayed are non-significant covariates (age, relationship length, number of ratings and understanding of symbols in the app). CI = 95%

### Moderation of accuracy and bias by experience with messengers and communication frequency

A moderation effect was found for experience with messengers on assumed similarity bias of partner’s valence. Contrary to our hypothesis, higher experience with messengers was related to higher reliance on own valence in the judgment of partner’s valence, as indicated by a significant interaction effect (see Table 4). A one-unit change in experience with messengers was related to a small increase in the influence of assumed similarity bias on the judgment of partner’s valence. A simple slope analysis of experience with messengers one standard deviation above (*B* = −0.065, *t* = −1.94, *p* = 0.0.056) and below (*B* = 0.229, *t* = 6.79, *p* < 0.001) the mean, revealed that only in the low experience with messenger condition there was a significant positive association between own valence and the judgment of partner’s valence. No moderation effects were found for tracking accuracy in the valence model. The same tendencies, but no statistically significant effects were found in the arousal model [see Table S3 in the Additional file 1]. Only age as a covariate had an impact in the valence and arousal model and no further statistically significant covariate effects were found.

**Table 4:** Moderation analysis: Mean-level bias, tracking accuracy, and projection bias in the judgment of partner’s valence and the moderation effect of experience with messengers

Effects	Estimate	*SE*	*t*	*p*	*CI*
Mean-level bias	−0.513	0.196	−2.61	0.012	[−0.906; −0.120]
Tracking accuracy	0.266	0.026	10.18	< .0001	[0.213; 0.319]
Projection bias	0.419	0.024	17.53	< .0001	[0.371; 0.468]
Experience with messengers	0.168	0.084	2.01	0.047	[0.002; 0.334]
Experience with messengers^X^ tracking accuracy	0.047	0.030	1.56	0.121	[−0.013; 0.107]
Experience with messengers^X^ projection bias	0.070	0.028	2.55	0.013	[0.016; 0.126]
Age	−0.253	0.057	−4.42	< .0001	[−0.368; −0.140]

*N* = 51 couples, 102 participants, 960 jointly reported ratings. Solution for fixed effects of multilevel truth and bias analysis. Estimates refer to unstandardized effects. Non-significant covariates (relationship length, number of ratings and understanding of symbols in the app) are not displayed. CI = 95%

^X^interaction effect

A moderation effect was found for messenger communication frequency on tracking accuracy of partner’s arousal of affect. As predicted, higher communication frequency was related to higher empathic accuracy, as indicated by a significant interaction effect (see Table 5). A one-unit change in communication frequency was associated with a small change in the influence of tracking accuracy on the judgment of partner’s arousal. A simple slope analysis of communication frequency one standard deviation above (*B* = −0.038, *t* = −1.49, *p* = 0.0.141) and below (*B* = 0.106, *t* = 4.39, *p* < 0.001) the mean revealed that only for low communication frequency there was significant positive association between the judgment of partner’s arousal and partner’s actual arousal. No moderation effects were found for assumed similarity bias in the arousal model. The same tendencies, but no statistically significant effects were found in the valence model [see Table S4 in the Additional file 1]. No covariate had a significant impact in the model, and the significant results remained after removing all covariates.

**Table 5:** Moderation analysis: Mean-level bias, tracking accuracy, and projection bias in the judgment of partner’s arousal and the moderation effect of communication frequency

Effects	Estimate	*SE*	*t*	*p*	*CI*
Mean-level bias	−0.020	0.028	−0.69	0.495	[−0.078; 0.039]
Tracking accuracy	0.090	0.025	3.66	0.001	[0.041; 0.140]
Projection bias	0.292	0.033	8.97	< .0001	[0.228; 0.358]
Communication frequency	0.029	0.047	0.62	0.538	[−0.065; 0.123]
Communication frequency^X^ tracking accuracy	0.076	0.034	2.27	0.027	[0.010; 0.144]
Communication frequency^X^ projection bias	0.023	0.045	0.52	0.607	[−0.067; 0.113]

*N* = 51 couples, 102 participants, 960 jointly reported ratings. Solution for fixed effects of multilevel truth and bias analysis. Estimates refer to unstandardized effects. CI = 95%. Non-significant covariates (age, relationship length, number of ratings, understanding of symbols in the app and use of additional messenger apps) are not displayed

^X^interaction effect

Post-hoc power analysis based on standardized regression estimates [see Table S5-S8 in the Additional file 1] using a dyadic data power calculation tool (APIMpower [71]) revealed a necessary number of 56 indistinguishable dyads for the effect of tracking accuracy in the full valence model and 477 dyads for the effect of tracking accuracy in the full arousal model with a power of 0.80, an alpha of 0.05 and identical actor and partner effects. 265 dyads were needed to detect the moderation effect of experience with messengers on similarity bias in the valence model and 1083 dyads were needed to detect the moderation effect of communication frequency on tracking accuracy in the arousal model.

## Discussion

The present research studied accuracy and bias in judgments of affect in couples’ messenger communication, using a novel messenger-based assessment method. We investigated to what extent judgments of partner’s valence and arousal of affect are in line with partner’s actual valence and arousal of affect. Furthermore, we tested for moderation effects of experience with messenger and messenger communication frequency. In general, we found evidence for tracking accuracy and bias of assumed similarity for both valence and arousal, a moderation effect of experience with messenger on bias of assumed similarity in the perception of partner’s valence, and a moderation effect of communication frequency on tracking accuracy in the perception of partner’s arousal. These results illustrate the interplay of accuracy and bias in couples’ judgment of each other in a text messaging environment. Furthermore, they demonstrate the utility of a novel messenger-based assessment method in interpersonal research.

### Messenger-based assessment of accuracy and bias in couples

Our analysis indicates a positive association between judgments of partner’s affect and partner’s actual affect in couples messenger communication. T&B analysis revealed that these associations are still present when general tendencies of perception bias or mean-level bias as well as the bias of assumed similarity with own affect are accounted for. These results are consistent with a range of studies that describe the interplay of both accuracy and bias in couples’ judgment of affect outside a smartphone environment [6, 44, 84]. The present study extends the ecological validity of these findings by using a novel messenger-based assessment method. To our knowledge, this is the first study to report the implementation of an assessment method, which extends the idea of event-contingent recordings [52] by reducing interference with events and minimizing delays between events and ratings. Analysis of the comprehension of symbols and procedures in the app, which was included as a covariate in all analysis, revealed that it had no impact on accuracy and bias. In sum, the feasibility of the method can be assumed.

The increment of methodological pluralism has been advocated for decades [50, 85, 86]. In diversifying assessments, including real-world and laboratory-based, the tradeoff between ecological or external and internal validity in interpersonal research can be accounted for [50]. Consequently, our research adds to the range of methodological approaches, which can broaden the perspective on couple interactions and increase the robustness of findings.

The technological development of assessment in couples has been addressed in various ways. Recent ideas focus on the development of inobtrusive observations, such as the computer-based coding of video recorded couple interactions [87] or the analysis of the content of couples’ text messages [88]. These developments could help to reduce the criterion problem in accuracy research, which describes the issue that the criterion with which the perception is compared (e.g. partner’s actual affect) might not be perfectly accessible to the partner and subject to bias [23]. The messenger-based approach on the other hand, involves active ratings of perception and criterion. However, we think this approach is useful in two ways. First, it enables researchers to avoid the collection of personally identifiable information or personal data. The separation of the data stream, which is relevant for the subsequent analysis, from the data stream of the actual interaction, which includes personal data, reduces the risk of security or privacy breaches and associated issues [89]. A recent review on privacy concerns argues that even when transparency and control, two central aspects of privacy, are given, insufficient levels of protection prevail [90]. Therefore, the messenger-based approach and our application of enrolment without personal data avoids issues related with observational methods of smartphone use, such as the application of screenshot apps [91] or mobile data donation [88]. Our approach is in line with privacy by design ideas that embed privacy and data protection in technologies itself, which is necessary in areas with highly sensitive private data [92]. The second advantage of our approach is that reciprocal ratings can be easily integrated in mobile interventions for couples. Indicating mental states and receiving feedback can be a cognitive method to train empathic accuracy [93] and the expression of affective information can increase empathic accuracy in couple conflicts [94]. Also, a messenger-based approach simplifies the development of mobile applications, which could improve the dissemination of interventions and prevention programs for couples [4, 95].

### Moderation of accuracy and bias by experience with messengers and communication frequency

The present study is, to our knowledge, the first to investigate the role of experience with messengers and communication frequency in couples’ accuracy and bias. As predicted, we found a significant moderation effect of couple communication frequency in messengers on accuracy of arousal judgments. These small effects, though being weak evidence, speak for general improvements in accuracy due to mere usage of texting. However, results were heterogenic with no significant impact of communication frequency on the judgment of partner’s valence. The differential effects for arousal and valence ratings could be explained by the fact, that arousal as a measure of behavioral activation is more closely linked to the frequency of interaction. Couples with higher levels of communication frequency could have more frequent cues for the behavioral activation and arousal level of the partner.

The analysis of the impact of participants’ experience with messengers on their empathic judgements revealed an effect that was contrary to our expectations. Partners with higher experience with messengers showed a stronger reliance on their own affective valence in empathic judgments of their partners’ affective valence. There was no significant effect on tracking accuracy in the valence of affect model and no moderation effect in the arousal of affect model, although similar patterns emerged. These findings contradicted our expectations of a lower reliance on own affective states with growing experience with messenger apps. These findings could be explained by a greater influence of other factors involved in empathic judgements. Knowledge of the partner is in some situations a reliable cue for the judgment of a partner’s affect, even when the partner is not present [96] and reliance on acquired knowledge could reduce the impact of perceiver’s own affect. In the context of messenger communication, partners with low experience for this modality could show a stronger reliance on general knowledge of their partner since it is constantly available in the rather uncertain context of text messages. In turn, this could result in a reduced reliance on the perceiver’s own affect or the bias of assumed similarity. Importantly, we found no changes in patterns when controlling for duration of relationship, which is in line with studies reporting no effect of relationship length on tracking accuracy [24]. Accuracy related general knowledge of the partner can be seen as mostly stable over time (see [23] for a meta-analysis). Furthermore, motivational aspects could explain the examined moderation effects of experience with messengers. Though we controlled for age in our analysis, motivational processes can play an important role in the compensation of empathic accuracy related cognitive performance in older adults and are shown to change patterns of empathic accuracy in older couples [97]. Much alike, participants with lower experience with messengers across all ages could have a higher motivation to correctly identify a partner’s affect, due to the novelty of the assessment method.

Inconsistencies in moderation effects between valence and arousal models, are in line with an earlier study comparing accuracy of valence and arousal [98] as well as studies on differences in the readability of valence and arousal [99, 100] and high rates of accuracy in the rating of valence of affect in strangers’ text messages [101]. Downstream analysis of moderation effects could be influenced by these differences, in the way that a higher communication frequency enables couples to identify the rather difficult to predict arousal level of a partner, which does not emerge in the valence model, since a partner’s valence is generally easier to judge. We used an affect grid with four arousal and seven valence categories to simplify the rating procedure and to fit the grid size to smartphone display proportion. Therefore, the assessment of arousal was not as fine-grained as valence ratings, which might have influenced the moderation analysis of experience with messengers. Though we checked for overall understanding of symbols in the app, differences in comprehending the concepts of valence and arousal might be present. In addition, the question of affect comprehension and conceptualization has been widely neglected in empathic accuracy research. It has been shown that individuals differ in the capability to experience, label [16], and differentiate [98] own affect, which are central processes for empathic accuracy [102]. In general, the discussed inconsistencies highlight the importance of a further elaboration of the criterion problem in empathic accuracy research.

### Strengths and limitations

Several limitations of our study must be mentioned. Our study is underpowered for most analysis, with our post-hoc power analysis showing that only the valence model had acceptable power of 0.76 to detect reported effect sizes. Although we found similar tendencies in valence and arousal models. Additionally, even though our hypotheses were preregistered, and main and exploratory hypothesis were differentiated in the preregistration, calculating multiple significance values without correcting for multiple comparisons is a limitation of the study. Interpretation of results, especially of the arousal model and the moderation analysis must be done with caution. Our sample consisted of German speaking couples living in Germany, so a low cultural diversity must be assumed. Cultural differences in systems of affect are widely studied (see [103] for a review) and couple interactions and its outcomes differ depending on culturally mediated affective processes and views of affect [104, 105]. Also, our study did not control for differences in socioeconomic status, which have been shown to influence dynamics and affective processes in couples [106]. Consequently, the generalizability of our findings to different cultures and socioeconomic status is reduced.

A mayor strength of our study is the inclusion of same-gender, mixed-gender, diverse gender couples, and participants, who did not indicate their gender. So far, most studies on empathic accuracy in couples focused on the term sex and relied on opposite-sex couples [3], which can be due to theoretical assumptions of differences in accuracy and bias in same-sex as opposed to opposite-sex couples [107] or a reliance on analytic strategies that only allow for distinguishable dyads with partners that are assignable to either of two distinct groups [78]. Though earlier studies have found that women’s accuracy has more severe consequences for outcomes in couples [3], differences between women and men regarding accuracy and bias are mixed [16, 108]. Our study is among the few to include diverse participants and participants, who did not state their gender and apply an appropriate statistical procedure for repeated-measures and indistinguishable dyads.

### Future research

The novel assessment method applied in the present study has not yet been compared to existing assessment methods of accuracy and bias in couples. In doing so, future studies could extend our understanding of different methodological problems, associated with couple research [109]. For example, to our knowledge, no study has yet thoroughly compared laboratory-based and real-life oriented data collection methods (for a combination of implicit and explicit methods see [110]).

We did not examine the impact of covert and disclosed ratings of affect in couples. It can be assumed that ratings over time with feedback, as in the case of disclosed ratings, can have an impact on accuracy and bias. The inclusion of training effects of empathic accuracy in couples can be a fruitful line of research with implications for couple interventions.

## Conclusions

Couples’ texting behavior is an important aspect of their daily interactions and associated with several changes in their reciprocal understanding. The present preregistered study is the first to use a messenger-based assessment procedure to examine couples’ accuracy and bias of affect. A smartphone messenger-based assessment approach might be able to overcome some limitations of earlier daily-life assessment methods. Consistent with our hypothesis we found that couples are accurate in their judgments of their partners’ affect in text messengers. Also, we found significant levels of projection bias, indicating that accuracy and bias shape partner judgments of affect in couples. We found a moderation effect of experience with messengers on bias of assumed similarity. It seems that higher experience with messengers is associated with a stronger reliance on own valence as a cue in the judgment of partner’s valence of affect. Finally, we found a moderation effect of communication frequency on tracking accuracy of partner’s arousal of affect, confirming that more communication via text messages was associated with higher accuracy in judgments.

## Supplementary Information

Additional file 1: Tables S1-S8. Additional results in table format

## Data Availability

The data that support the findings of this study are not openly available due to reasons of data privacy and protection. Due to the sensitivity of the data, the high level of indirect identifiers in dyadic couple data, and the novelty of the assessment procedure, informed consent was not obtained for publication of participant data. Data are available from the corresponding author upon reasonable request and with the permission of Ethics Committee at Ethics Committee of the Faculty of Medicine, University Goettingen. Data are located in controlled access data storage at University Computing Centre of the University Goettingen (GWDG).

## Footnotes

1 The present study addresses hypothesis 4.1 of the preregistration [66]. See [67] for the investigation of Hypotheses 4.2 and 4.3.

2 Preregistered power calculations were also conducted using APIMPower by Kenny and Ackermann [71] based on the meta-analytical effect size for the relation between empathic accuracy and relationship satisfaction (r = .134 [3]), a desired power of .08, alpha of .05 and indistinguishable dyads, resulting in a necessary sample size of 198 dyads.

3 Messenger-based assessment also included ratings of interpersonal motives (one rating for self, one rating for partner), evaluated using an interpersonal circumplex model of agency and communion. The present study focuses on ratings of affect and results of hypotheses on interpersonal motives are currently prepared for publication.

4 Contrary to our procedure, we preregistered the person-mean centering of outcome, tracking accuracy and bias. However, as the present analysis is interested in general effects, as opposed to effects on the person-level, we applied grand-mean centering following Kenny, Kashy, & Cook [78].

5 SAS analysis script and full model specifications can be found in the online supplement [68].

6 The model without covariates showed significant mean-level bias for the valence model. No differences were found for the arousal model [see Table S1 and S2 in Additional file 1].
