# Supplementary material for: Messenger-based assessment of empathic accuracy in couples’ smartphone communication
Source: BMC Psychol. 2025 Feb 21;13:147. doi: 10.1186/s40359-025-02483-9 (PMC11844058; doi:10.1186/s40359-025-02483-9)
Supplement: Supplementary file 1 — Additional file 1: Tables S1-S8. Additional results in table format [file 40359_2025_2483_MOESM1_ESM.docx]

# Online Supplementary Material

**Table S1**

*General mean-level bias, tracking accuracy and projection bias in the judgment of partner’s valence without covariates.*

| Effects | Estimate | *SE* | *t* | *p* | *CI* |
| --- | --- | --- | --- | --- | --- |
| Mean-level bias | -0.332 | 0.116 | -2.86 | 0.006 | [-0.565; -0.099] |
| Tracking accuracy | 0.264 | 0.027 | 9.94 | <.0001 | [0.210; 0.317] |
| Projection bias | 0.418 | 0.026 | 15.80 | <.0001 | [0.365; 0.472] |

*Note. N* = 51 couples, 102 participants, 960 jointly reported ratings. Solution for fixed effects of multilevel truth and bias analysis. Estimates refer to unstandardized effects. CI = 95%.

**Table S2**

*General mean-level bias, tracking accuracy and projection bias in the judgment of partner’s arousal without covariates.*

| Effects | Estimate | *SE* | *t* | *p* | *CI* |
| --- | --- | --- | --- | --- | --- |
| Mean-level bias | -0.026 | 0.026 | -1.01 | 0.318 | [-0.079; 0.027] |
| Tracking accuracy | 0.086 | 0.026 | 3.35 | 0.001 | [0.035; 0.137] |
| Projection bias | 0.286 | 0.032 | 8.89 | <.0001 | [0.222; 0.350] |

*Note. N* = 51 couples, 102 participants, 960 jointly reported ratings. Solution for fixed effects of multilevel truth and bias analysis. Estimates refer to unstandardized effects. CI = 95%.

**Table S3**

*Moderation analysis: General mean-level bias, tracking accuracy and projection bias in the judgment of partner’s arousal and the moderation effect of experience with messengers.*

| Effects | Estimate | *SE* | *t* | *p* | *CI* |
| --- | --- | --- | --- | --- | --- |
| Mean-level bias | -0.513 | 0.196 | -2.61 | 0.012 | [-0.906; -0.120] |
| Tracking accuracy | 0.266 | 0.026 | 10.18 | <.0001 | [0.213; 0.319] |
| Projection bias | 0.419 | 0.024 | 17.53 | <.0001 | [0.371; 0.468] |
| Experience with messengers | 0.168 | 0.084 | 2.01 | 0.047 | [0.002; 0.334] |
| Experience with messengers*  tracking accuracy | 0.047 | 0.030 | 1.56 | 0.121 | [-0.013; 0.107] |
| Experience with messengers*  projection bias | 0.070 | 0.028 | 2.55 | 0.013 | [0.016; 0.126] |
| Age | -0.253 | 0.057 | -4.42 | <.0001 | [-0.368; -0.140] |

*Note. N* = 51 couples, 102 participants, 960 jointly reported ratings. Solution for fixed effects of multilevel truth and bias analysis. Estimates refer to unstandardized effects. Not displayed are non-significant covariates (relationship length, number of ratings and understanding of symbols in the app). CI = 95%.

**Table S4**

*Moderation analysis: General mean-level bias, tracking accuracy and projection bias in the judgment of partner’s valence and the moderation effect of communication frequency.*

| Effects | Estimate | *SE* | *t* | *p* | *CI* |
| --- | --- | --- | --- | --- | --- |
| Mean-level bias | -0.415 | 0.226 | -1.83 | 0.071 | [-0.867; 0.037] |
| Tracking accuracy | 0.266 | 0.028 | 9.67 | <.0001 | [0.211; 0.322] |
| Projection bias | 0.422 | 0.027 | 15.72 | <.0001 | [0.368; 0.477] |
| Communication frequency | 0.137 | 0.128 | 1.07 | 0.286 | [-0.117; 0.391] |
| Communication frequency*  tracking accuracy | 0.017 | 0.038 | 0.46 | 0.651 | [-0.060; 0.094] |
| Communication frequency*  projection bias | 0.040 | 0.037 | 1.06 | 0.293 | [-0.036; 0.115] |
| Age | -0.283 | 0.057 | -4.92 | <.0001 | [-0.397; -0.169] |

*Note. N* = 51 couples, 102 participants, 960 jointly reported ratings. Solution for fixed effects of multilevel truth and bias analysis. Estimates refer to unstandardized effects. Not displayed are non-significant covariates (relationship length, number of ratings, understanding of symbols in the app and use of other messenger apps). CI = 95%.

**Table S5**

*Standardized effects: General mean-level bias, tracking accuracy and projection bias in the judgment of partner’s valence.*

| Effects | Estimate | *SE* | *t* | *p* | *CI* |
| --- | --- | --- | --- | --- | --- |
| Mean-level bias | 0.001 | 0.066 | 0.02 | 0.984 | [-0.133; 0.136] |
| Tracking accuracy | 0.248 | 0.026 | 9.40 | <.0001 | [0.195; 0.302] |
| Projection bias | 0.402 | 0.025 | 15.98 | <.0001 | [0.351; 0.453] |
| Age | -0.210 | 0.043 | -4.88 | <.0001 | [-0.296; -0.125] |

*Note. N* = 51 couples, 102 participants, 960 jointly reported ratings. Solution for fixed effects of multilevel truth and bias analysis. Not displayed are non-significant covariates (relationship length, number of ratings and understanding of symbols in the app). Estimates refer to standardized effects (model with z-standardized variables). CI = 95%.

**Table S6**

*Standardized effects: General mean-level bias, tracking accuracy and projection bias in the judgment of partner’s arousal.*

| Effects | Estimate | *SE* | *t* | *p* | *CI* |
| --- | --- | --- | --- | --- | --- |
| Mean-level bias | 0.019 | 0.033 | 0.56 | 0.578 | [-0.05; 0.087] |
| Tracking accuracy | 0.089 | 0.026 | 3.39 | 0.001 | [0.037; 0.141] |
| Projection bias | 0.294 | 0.033 | 8.97 | <.0001 | [0.229; 0.359] |

*Note. N* = 51 couples, 102 participants, 960 jointly reported ratings. Solution for fixed effects of multilevel truth and bias analysis. Estimates refer to standardized effects (model with z-standardized variables). Not displayed are non-significant covariates (age, relationship length, number of ratings and understanding of symbols in the app). CI = 95%.

**Table S7**

*Moderation analysis of standardized effects: General mean-level bias, tracking accuracy and projection bias in the judgment of partner’s valence and the moderation effect of experience with messengers.*

| Effects | Estimate | *SE* | *t* | *p* | *CI* |
| --- | --- | --- | --- | --- | --- |
| Mean-level bias | -0.347 | 0.304 | -1.14 | 0.259 | [-0.955; 0.262] |
| Tracking accuracy | 0.404 | 0.045 | 8.93 | <.0001 | [0.313; 0.496] |
| Projection bias | 0.717 | 0.042 | 17.26 | <.0001 | [0.633; 0.801] |
| Experience with messengers | 0.299 | 0.128 | 2.33 | 0.022 | [0.045; 0.554] |
| Experience with messengers*  tracking accuracy | 0.068 | 0.051 | 1.34 | 0.182 | [-0.033; 0.169] |
| Experience with messengers*  projection bias | 0.116 | 0.049 | 2.38 | 0.020 | [0.019; 0.214] |
| Age | -0.353 | 0.113 | -3.13 | 0.002 | [-0.577; -0.129] |

*Note. N* = 51 couples, 102 participants, 960 jointly reported ratings. Solution for fixed effects of multilevel truth and bias analysis. Estimates refer to standardized effects (model with z-standardized variables). Not displayed are non-significant covariates (relationship length, number of ratings and understanding of symbols in the app). CI = 95%.

**Table S8**

*Moderation analysis of standardized effects: General mean-level bias, tracking accuracy and projection bias in the judgment of partner’s arousal and the moderation effect of communication frequency.*

| Effects | Estimate | *SE* | *t* | *p* | *CI* |
| --- | --- | --- | --- | --- | --- |
| Mean-level bias | 0.021 | 0.034 | 0.60 | 0.553 | [-0.05; 0.091] |
| Tracking accuracy | 0.091 | 0.025 | 3.66 | 0.001 | [0.042; 0.142] |
| Projection bias | 0.296 | 0.033 | 8.97 | <.0001 | [0.231; 0.362] |
| Communication frequency | 0.026 | 0.041 | 0.62 | 0.538 | [-0.057; 0.108] |
| Communication frequency*  tracking accuracy | 0.056 | 0.025 | 2.27 | 0.027 | [0.007; 0.106] |
| Communication frequency*  projection bias | 0.017 | 0.033 | 0.52 | 0.607 | [-0.049; 0.083] |

*Note. N* = 51 couples, 102 participants, 960 jointly reported ratings. Solution for fixed effects of multilevel truth and bias analysis. Estimates refer to standardized effects (model with z-standardized variables). Not displayed are non-significant covariates (age, relationship length, number of ratings, understanding of symbols in the app). CI = 95%.

**Figure S1**

*Rating interface int the messenger-based assessment app.*


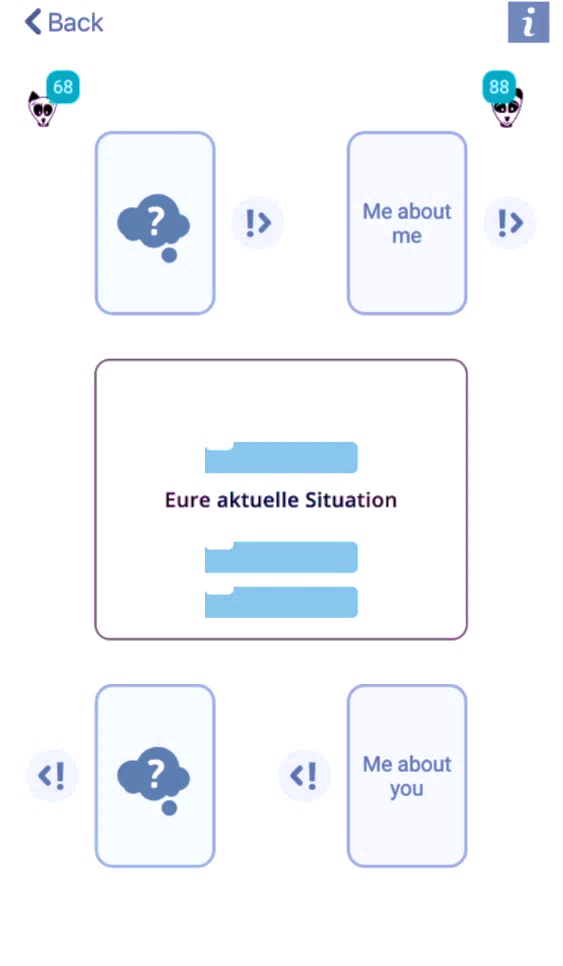


**Current chat**

*Note.* Rating interface in the smartphone messenger app. Own affect can be indicated in the top right corner and rating of partner’s affect can be indicated in the lower right corner. The center indicates the context of the assessment situation. The assessment is completed by clicking on the button on the screen and selecting the respective rating in the assessment matrix.

**Figure S2**

*Messenger-based Assessment matrix of affect*


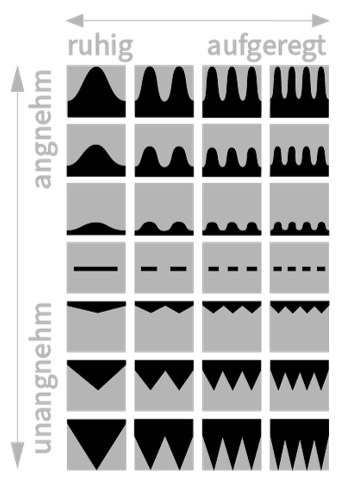


**Negative Valence Positive Valence**

**Low Arousal High Arousal**

*Note.* Rating matrix of affect in the smartphone messenger app is based on the valence-arousal model of affect by Posner, Russel, and Peterson [1]. Shape of waves indicate valence and number of waves indicate arousal.

**English language version of questionnaire measures**

**Age**

How old are you?

- Under 18
- 18-25
- 26-30
- 31-35
- 36-40
- 41-45
- 46-50
- 51-55
- 56-60
- 61-65
- Over 65
- Not specified

**Relationship length**

How long have you been in a relationship with your partner?

- Less than 1 year
- 1-3 years
- 4-10 years
- More than 10 years
- Not specified

**Experience with messengers**

How experienced are you personally with the usage of messenger apps?

Very little (1) - - - very much (5)

**Understanding of symbols in the app**

I understood the content of the app well.

Do not agree at all (1) - - neither nor (4) - - agree completely (7) (not specified)

**Use of other messenger apps**

During the study, I used other messenger apps to communicate with my partner.

Do not agree at all (1) - - neither nor (4) - - agree completely (7) (not specified)

**References**

1. Posner J, Russell JA, Peterson BS. The circumplex model of affect: An integrative approach to affective neuroscience, cognitive development, and psychopathology. Dev Psychopathol. 2005;17:715–34. doi:10.1017/S0954579405050340.
